# Sudden onset of static equilibrium dysfunction in patients receiving a cochlear implant

**DOI:** 10.1097/MD.0000000000008465

**Published:** 2017-11-03

**Authors:** Ying Gao, Qing Zhang, Jing Yan, Xiaorong Niu, Peng Han, Haifeng Yuan, Juan Hu, Bo Liu, Min Xu

**Affiliations:** a Department of Otorhinolaryngology Head and Neck Surgery, Ear Institute, Second Affiliated Hospital, Xi’an Jiaotong University College of Medicine, Xi’an, Shaanxi Province; b Department of Otorhinolaryngology Head and Neck Surgery, Ear Institute, First Affiliated Hospital, Xi’an Jiaotong University College of Medicine, Xi’an, Shaanxi Province; c Department of Otolaryngology, Union Hospital, Tongji Medical College, Huazhong University of Science and Technology, Wuhan, Hebei Province, P.R. China.

**Keywords:** Dizziness, postural control, vestibular plasticity

## Abstract

**Background:**

We investigated the sudden onset of static equilibrium dysfunction caused by cochlear implantation (CI) in congenital hearing loss patients.

**Method:**

Twenty-five patients were selected from a cohort of unilateral CI recipients to form the CI group. Static posturography was performed 1 to 3 days before and 3 to 5 days after CI. Each patient underwent the test with eyes open (EO) and eyes closed (EC) for 30 seconds, separately. Another group of age- and sex-matched patients with no history of hearing impairment undergoing unrelated surgeries formed the control group, and were examined with the same tests pre- and post-surgery. A third group of patients undergoing middle ear surgery formed the otitis media (OM) group. Postural sway parameters including sway velocity (SV) in the *X*-axis, SV in the *Y*-axis, length of sway locus length (LNG), and environmental area (ENV) were measured and recorded.

**Results:**

Comparison of pre-surgery posturographical parameters between the CI and control groups revealed no significant differences. Significant differences were found in most parameters in pre- and post-surgery comparisons in the CI group. Mean SV values in the *X*-axis pre- and post-surgery were 8.48 and 11.52 mm/s, respectively, in the EO condition (*P* < .05), and 14.94 and 20.16 mm/s, respectively, in the EC condition (*P* < .05). In the *Y*-axis, mean SV values were 15.36 and 20.24 mm/s pre- and post-surgery, respectively, in the EC condition (*P* < .05). The LNG values in the CI group pre- and post-surgery were 319.60 and 469.88 mm in the EO condition (*P* < .05), and 571.40 and 764.12 mm in the EC condition (*P* < .05). No significant functional equilibrium change was observed in the control group between pre- and post-surgery (*P* > .05) except SV in the *X*-axis and LNG in the EO condition (*P* < .05). No significant pre- and post-surgery differences were found in the OM group.

**Conclusion:**

CI appeared to influence static equilibrium function within 1 week post-surgery. This influence was greater when eyes were closed.

## 1 Introduction

Cochlear implantations (CIs) are commonly used worldwide to help deaf people develop language skills, especially in children with bilateral severe sensorineural hearing loss. Together with the cochlea (part of the auditory system), the vestibular system constitutes the labyrinth of the inner ear. Because the vestibular end organs and the cochlea are closely related, impairments of the cochlear and vestibular systems are often associated. Previous studies have reported that CI surgery can have a significant negative effect on the results of caloric as well as vestibular evoked myogenic potential (VEMP) tests, whereas no significant effect of CI surgery was detected in the head impulse test (HIT).^[1–5]^ However, these tests only reflect the vestibular functional inputs from the visual system and the vestibular organs in the ear.

Posturography is a set of tests for assessing the integrative vestibular performance associated with the maintenance of posture, which involves integration between the vestibular system and other sensory inputs, such as vision and proprioception. A method for equilibrium function evaluation was developed by Nashner et al^[6,7]^ and has been in commercial use since 1986.

A small number of previous studies have examined posturography in relation to CI. Buchman^[8]^ assessed the vestibular function of CI recipients 1 month after surgery using dynamic platform posturography. The results revealed substantial improvement in postural sway in patients with vestibular conditions, both with the device “off” and “on.” In clinical practice, it was found that most patients, particularly children, exhibited postural imbalance within 1 week after CI, with some patients complaining of dizziness, vertigo, and balance impairment.^[9]^ In the present study, we sought to verify whether CI affects balance within 1 week of CI surgery. Thus, we used static posturography to assess 25 CI recipients’ equilibrium functioning pre- and post-surgery, evaluating the sudden onset of static equilibrium dysfunction caused by CI in congenital hearing loss patients. To exclude the involvement of visual input, the test was performed in EO and EC conditions separately.

## 2 Material and methods

### 2.1 Patients

A test population (n = 25) was selected from a cohort of unilateral CI recipients at the Second Affiliated Hospital of Xi’an Jiaotong University from June 2014 to December 2014 to form the CI group (mean age 12.64 ± 4.22 years; range 6–27 years, female/male 11/14). According to medical records, each patient in the CI group suffered from hearing loss since birth, or exhibited an extremely poor response to sound since childhood. The CI was placed in the right ear of 21 patients and in the left ear of 4 patients.

All CI group patients fulfilled the following inclusion criteria: congenital bilateral profound sensorineural hearing loss; no history of other ear disorders; intact eardrums and normal middle ear pressure confirmed by tympanogram; normal inner ear structure; and no history of neurological disorders. Surgery in the CI group was performed using the round window approach after a regular mastoidectomy with posterior tympanotomy. Before opening the round window, bone dust and pate must be thoroughly irrigated away to avoid their entry into the cochlea or contact with the electrode array. In addition, the round window niche must be opened with a low revolving speed to avoid trauma caused by the shaking of endolymphatic fluid on the basilar membrane. The surgeon must take precautions when suctioning the perilymph, because the suction tip can cause mechanical damage to the basilar membrane and osseous spiral lamina. The insertion must then be performed with as little pressure as possible.^[10]^ In the present surgeries, we used Austrian Combi40 electrodes. In addition to the CI group, we selected 24 patients as a control group (mean age 12.58 ± 7.02 years; range 5–27 years, male/female 13/11), who fulfilled the following inclusion criteria: age- and sex-matched with the CI group patients; underwent general anesthesia without otology surgery in the department; normal hearing without current or past hearing-related medical diagnosis; no symptoms of OM; and no injuries affecting equilibrium. The control group was composed of 8 adenoid hypertrophy patients, 2 neck mass patients, 4 chronic sinusitis patients, and 10 chronic tonsillitis patients. Patients in both groups were narcotized using the same anesthetic. No patients in either group complained of dizziness or equilibrium dysfunction before the surgery. The surgery took approximately 1 hour in each case. Patients in both groups underwent surgery under general anesthesia. Demographic data of both groups are shown in Table 1.

**Table 1:**
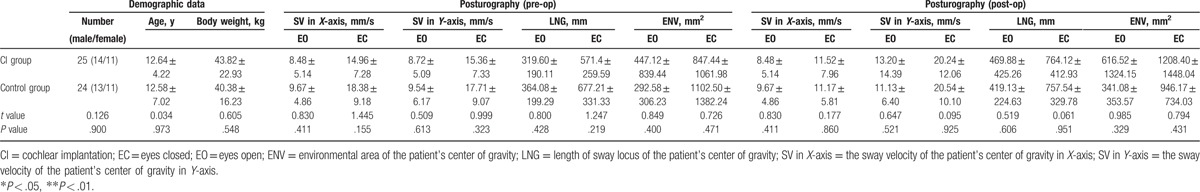
Comparison between CI group and control group.

In addition to the CI and control groups, we recruited an OM group, consisting of 16 patients with chronic suppurative OM and 12 patients with cholesteatoma OM unilaterally (mean age 41.00 ± 15.35 years; range 11–67 years, male/female 12/16). All OM patients underwent computed tomography (CT) scanning before surgery to confirm that the focus did not affect the inner ear. None of the OM group patients complained of vertigo before surgery.

### 2.2 Static posturography

The test equipment (Tecnobody PK254, Italy) included a computer to record the position and movement of the gravity center of the human body. Each patient stood on a platform, keeping the body as stable as possible. The position and movement were then recorded in EO and EC conditions, for 30 seconds in each condition. During EO, the visual system, proprioceptive system, and vestibular system inputs are involved in equilibrium adjustment. During EC, visual system input is deprived. Thus, only the proprioceptive system and vestibular system are involved in equilibrium function. All data were recorded for 30 seconds. Four parameters (SV in the *X*-axis, SV in the *Y*-axis, LNG, and ENV) were measured and used as indicators of postural stability.

The static equilibrium function was tested before and after surgery. The test was performed at the same scheduled time on the same day in all 3 groups. The study was approved by the Second Affiliated Hospital of Xi’an Jiaotong University ethical committee (2014115). Informed consent was obtained from all patients recruited in the study.

### 2.3 Statistical analysis

Paired *t*-tests were used to evaluate the difference between pre- and post-surgery data. Independent *t*-tests were used to evaluate the mean differences in SV values in the X and Y axes, as well as differences in LNG and ENV between the CI and control groups. Differences were considered statistically significant when the *P* value was <.05. Statistical analysis was performed using the Statistical Package for the Social Sciences (SPSS 10.0, International Business Machines Corporation, Xi’an Shaanxi, P.R. China).

## 3 Results

### 3.1 Comparison between the CI and control groups

The results are shown in Table 1. No statistically significant differences were found in patient number, age, or body weight between the CI and control groups (*P* < .05). No significant differences were found in any of the 4 parameters (SV in *X*-axis, SV in *Y*-axis, LNG, and ENV) between the CI and control groups pre-surgery (in both EO and EC conditions, *P* > .05), or post-surgery (in both EO and EC conditions, *P* > .05). No patients in the CI and control groups complained of equilibrium problems within 1 week after the operation.

### 3.2 Pre- and post-surgery comparison in the CI group

A significant difference was found between pre- (8.48 ± 5.14 mm/s) and post-surgery (11.52 ± 7.96 mm/s) in SV of *X*-axis values in the EO condition in the CI group (*P* < .05). Similarly, there was a significant difference between pre- (14.96 ± 7.28 mm/s) and post-surgery (20.16 ± 10.49 mm/s) in SV of *X*-axis values in the EC condition (*P* < .05), as well as in SV of *Y*-axis in the EC condition (pre- 15.36 ± 7.33 mm/s, post- 20.24 ± 12.06 mm/s, *P* < .01). A significant difference was found between pre- (319.60 ± 190.11 mm) and post-surgery (469.88 ± 425.26 mm) in LNG values of the CI group in the EO condition (*P* < .05), and a greater difference was found in the EC condition (pre- 571.40 ± 259.59 mm, post- 764.12 ± 412.93 mm, *P* < .01). No significant differences were found between pre- (8.72 ± 5.09 mm/s) and post-surgery (13.20 ± 14.39 mm/s) in SV of *Y*-axis values of the CI group in the EO condition (*P* > .05), or in ENV values in the EO condition (pre- 447.12 ± 839.44 mm^2^, post- 616.52 ± 1324.15 mm^2^, *P* > .05) or in the EC condition (pre- 847.44 ± 1061.98 mm^2^, post- 1208.40 ± 1448.04 mm^2^, *P* > .05).

### 3.3 Pre- and post-surgery comparison in the control group

Significant differences were only found between pre- (9.67 ± 4.86 mm/s) and post-surgery (11.17 ± 5.81 mm/s) in SV of *X*-axis values in the EO condition (*P* < .05), and between pre- (364.08 ± 199.29 mm) and post-surgery (419.13 ± 224.63 mm) in LNG values in the EO condition (*P* < .05). No significant differences were found in a comparison between pre- (18.38 ± 9.18 mm/s) and post- (19.75 ± 7.93 mm/s) in SV in *X*-axis in the EC condition, between pre- (9.54 ± 6.17 mm/s) and post- (11.13 ± 6.40 mm/s) in SV in *Y*-axis in the EO condition (*P* > .05). Similarly, no significant differences were found in SV in *Y*-axis values in the EC condition, or in LNG values in the EC condition (*P* > .05). In addition, no significant differences were found in ENV values in either the EO or EC conditions.

### 3.4 Pre- and post-surgery comparison in the OM group

Comparison between pre- and post-surgery in the OM group revealed no significant differences in any of the 4 parameters, in either the EO or EC conditions (*P* > .05).

**Figure 1:**
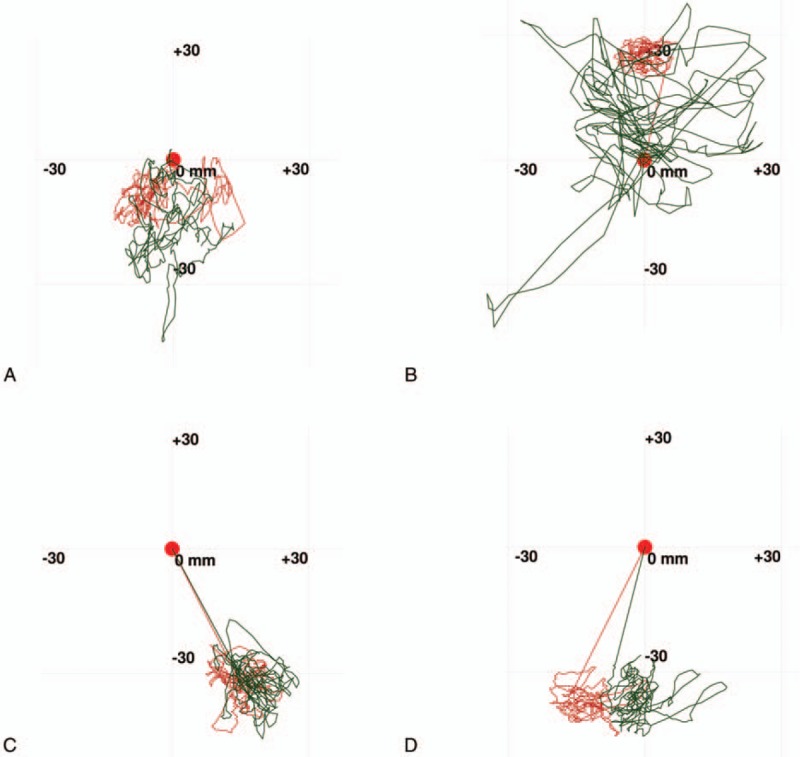
(A) Typical LNG in CI group pre-surgery. (B) Typical LNG in CI group post-surgery. (C) Typical LNG in control group pre-surgery. (D) Typical LNG in control group post-surgery. The green line shows the sway locus in the EO condition. The red line shows the sway locus in the EC condition. CI = cochlear implantation, LNG = length of sway locus.

**Figure 2:**
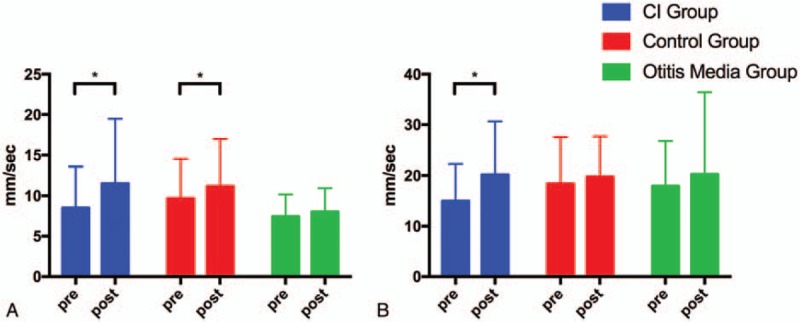
Pre- and post-surgery comparison of SV in the *X*-axis in the CI group, control group and OM group. (A) EO condition. (B) EC condition. CI = cochlear implantation, EC = eyes closed, EO = eyes open, OM = otitis media, SV = sway velocity. ^∗^*P* < .05; ^∗∗^*P* < .01.

**Figure 3:**
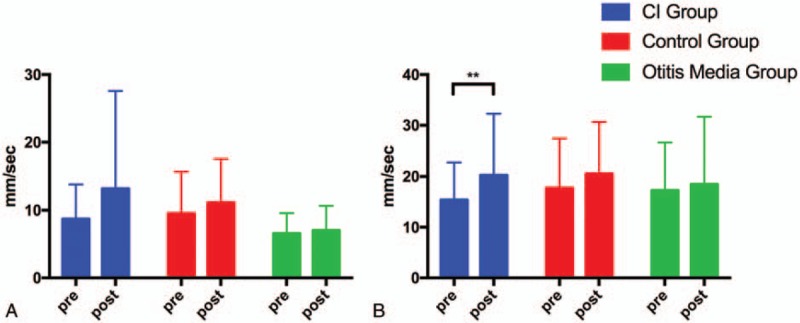
Pre- and post-surgery comparison of SV in the *Y*-axis in the CI group, control group, and OM group. (A) EO condition. (B) EC condition. SV in the *Y*-axis, and LNG and ENV in the EO and EC conditions. CI = cochlear implantation, EC = eyes closed, EO = eyes open, ENV = environmental area, LNG = length of sway locus, OM = otitis media, SV = sway velocity. ^∗^*P* < .05; ^∗∗^*P* < .01.

**Figure 4:**
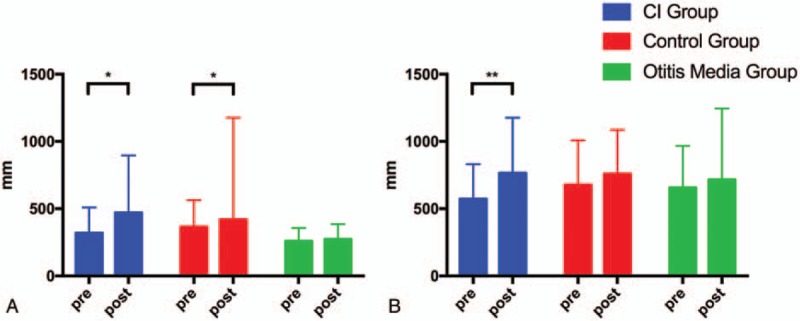
Pre- and post-surgery comparison of LNG in the CI group, control group, and OM group. (A) EO condition. (B) EC condition. CI = cochlear implantation, EC = eyes closed, EO = eyes open, OM = otitis media. ^∗^*P* < .05; ^∗∗^*P* < .01.

**Figure 5:**
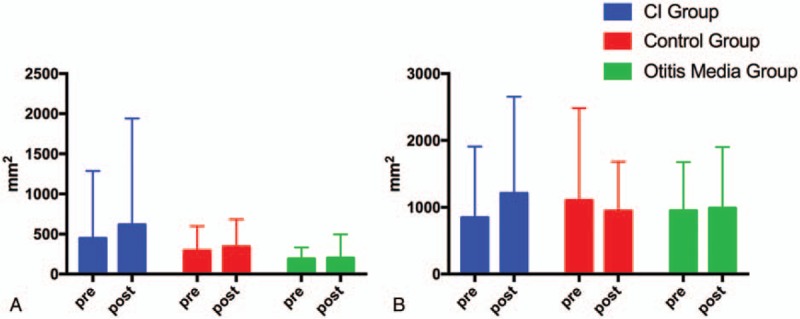
Pre- and post-surgery comparison of ENV in the CI group, control group, and OM group. (A) EO condition. (B) EC condition. EC = eyes closed, EO = eyes open, ENV = environmental area, LNG = length of sway locus. ^∗^*P* < .05; ^∗∗^*P* < .01.

Figure 1 shows the typical sway locus length pre- and post-surgery in the CI and control groups. According to the patients’ medical records, significant differences were present between the CI and control group values pre- and post-operation. The results of the pre- and post-surgery comparison in the 3 groups are shown in Table 2 and Figures 2–5.

**Table 2:**
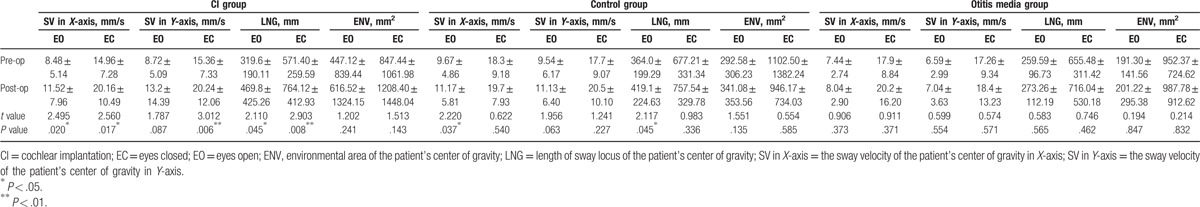
Comparison between pre-op and post-op in CI group, control group, and otitis media group.

## 4 Discussion

Patients with vestibular loss show changes in visual, somatosensory, and vestibular organs; central nervous system processing; and coordination of the musculoskeletal system, all of which are involved in the maturation of equilibrium function. According to previous studies, adolescents with long-term use of CIs experience dysfunction of static equilibrium function.^[11,12]^ Several previous reports have examined the effects of CI devices (switched “on” and “off”) on the vestibular system.^[13,14]^ One study used histopathological analysis to examine 11 pairs of temporal bones after unilateral CI insertion, finding significant damage to the end organs of 6 patients, causing fibrosis in the vestibule, saccule membrane distortion, new bone formation, and reactive neuromas 1 to 17 years post-surgery.^[15]^ Moreover, some CI patients have been reported to suffer from acute, short-term, in most cases transient vertigo after CI surgery. Acute equilibrium injury is directly associated with vestibular dysfunction, which could be caused by surgical trauma during CI implantation. However, few studies have examined vestibular and equilibrium functions after CI.

To our knowledge, the present study provides the first report of the onset of static equilibrium dysfunction within 1 week of CI. The results revealed that CI patients showed poor post-surgery equilibrium function compared with pre-surgery, particularly in the EC condition. Without deprivation of any sensory input (visual, vestibular or proprioceptive system inputs), post-surgery performance was slightly worse than pre-surgery in the CI group. In addition, in the EC condition, the SV in the *Y*-axis and the LNG were significantly higher post-surgery compared with pre-surgery. The somatosensory and vestibular organs are responsible for the stabilization of equilibrium function. Patients with vestibular impairment typically show developmentally delayed gross motor skills, such as delayed head control, sitting, and walking. Importantly, the degree of vestibular loss is not proportionate to the degree of equilibrium function loss. This phenomenon may be due to compensation mechanisms. The vestibular system sends signals primarily to the brain and cerebellum that control eye movements, providing the anatomical basis of the vestibulo-ocular reflex, and to the muscles that control posture, which are necessary for maintaining an upright position. The brain uses information from the vestibular system in the head and from proprioception throughout the body to understand the body's dynamics and kinematics, including position, balance, movement, and acceleration. The cerebellum is largely responsible for coordinating the unconscious aspects of proprioception. When one of the inputs is dysfunctional, the others will undergo adaptive changes via compensatory mechanisms.

A number of possible mechanisms may be involved in the sudden onset of static equilibrium dysfunction caused by CI. Surgical trauma: the vibration caused by drilling the cochlea might dislodge otoconia into the labyrinth, and bone dust particles might fall into the cochlea, both of which could cause the onset of benign paroxysmal positional vertigo (BPPV) after CI insertion.^[16]^ Insertional trauma: insertion of the CI could damage the osseous spiral lamina, basilar membrane, and vestibular receptors.^[17,18]^ Intraoperative loss of perilymph fluid,^[19]^ acute serous labyrinthitis due to cochleostomy, implant and bodily reaction to labyrinthitis, endolymphatic hydrops, and electrical stimulation by the implant.^[20,21]^

Subjects in the control group showed worse post-surgery equilibrium function compared with pre-surgery, as shown by the SV results in the *X*-axis and LNG results under EO condition. This effect might have been due to the influence of the intravenous anesthetic. Although the drugs used for general anesthesia did not directly influence vestibular and equilibrium function, and possessed a half-life period of <24 hours, the influence of general anesthetic should be considered. In addition, the patients were debilitated, with reduced physical strength after the surgery. These factors represent potential confounding factors that may have influenced the current results.

In the OM group, subjects showed no equilibrium problem post-surgery, compared with pre-surgery situation, in either the EO or the EC condition. This finding indicates that middle ear surgery, including mastoidectomy and tympanoplasty, did not influence patients’ balance functioning.

Comparison of SV, LNG, and ENV between the CI and control groups pre-surgery (in both the EO and EC conditions) showed no significant differences, suggesting that patients with congenital hearing loss exhibited no dysfunction on equilibrium. Previous studies conducted neurotologic interviews in 1003 subjects, finding lifetime adult prevalence of vestibular vertigo of 7.4%, and 1-year prevalence of 4.9%.^[22,23]^ Moreover, vestibular vertigo is reported to be 3 times more common among older people, and almost 3 times more common among females.^[24,25]^

The present study involved several limitations that should be considered. First, the study did not include a condition in which subjects stood on a foam pad in the EO and EC conditions. When standing on a foam pad, the proprioceptive system is deprived to the greatest extent, meaning that the vestibular system can be observed to the greatest extent when a subject is standing on a foam pad in the EC condition. Future studies should include a foam pad condition to examine this issue. Another limitation of the present study was the lack of analysis of different deafness etiologies. The deafness etiology in the CI patients included enlarged vestibular aqueduct, cochlear hypoplasia, possible congenital infection, and other unknown causes. However, the sample size for each etiology was too small to perform a subgroup analysis. Further analysis should be performed in a larger study population to take deafness into consideration in future studies.

## 5 Conclusion

CI appeared to influence static equilibrium function within 1 week post-surgery. This influence was greater when eyes were closed.

## Acknowledgment

We thank Benjamin Knight, MSc, from Liwen Bianji, Edanz Editing China (www.liwenbianji.cn/ac), for editing the English text of a draft of this manuscript.
